# Acute febrile illness in remote and rural communities: Perspectives of health providers and managers in Chattogram, Bangladesh

**DOI:** 10.1016/j.ssmhs.2025.100090

**Published:** 2025-12

**Authors:** Marco Liverani, Sharmin Ahmed, Aninda Sen, Rusheng Chew, Arjun Chandna, Shayla Islam, Richard J. Maude, Akramul Islam, Yoel Lubell

**Affiliations:** a London School of Hygiene and Tropical Medicine, London, UK; b Mahidol Oxford Tropical Medicine Research Unit, Bangkok, Thailand; c Centre for Tropical Medicine and Global Health, University of Oxford, Oxford, UK; d Faculty of Medicine, University of Queensland, Brisbane, Australia; e Cambodia Oxford Medical Research Unit, Angkor Hospital for Children, Siem Reap, Cambodia; f Amsterdam Institute for Global Health and Development, Amsterdam, the Netherlands; g School of Tropical Medicine and Global Health, Nagasaki University, Nagasaki, Japan; h Faculty of Public Health, Mahidol University, Bangkok, Thailand; i The Open University, Milton Keynes, UK; j Karolinska Institute, Erasmus Mundus, Solna, Sweden; k Virginia Commonwealth University, Richmond, VA, United States; l BRAC Health Programme, Dhaka, Bangladesh

**Keywords:** Bangladesh, Acute febrile illness, Community health workers, Referral system, Healthcare access in remote and rural areas

## Abstract

Acute febrile illness is a major public health issue in tropical regions, particularly in remote and rural areas where healthcare access is difficult. In these areas, patients often seek care too late or rely on self-medication, leading to increased risks of complications and death. This qualitative study examined challenges and opportunities for managing acute febrile illness in Chattogram Division, Bangladesh. The research involved interviews with 35 stakeholders across the health sector in Bangladesh, including health managers and frontline health workers, and international experts. Findings show that referral barriers, health seeking behaviour and financial considerations influence case management and outcomes in complex ways. Community health workers are vital in managing local health issues, but they have limited ability to address the evolving concerns about febrile illness. Gaps in the referral system and inadequate emergency care resources further complicate the situation. Many episodes of fever remain undifferentiated and are often managed with presumptive antibiotic treatment, which may be inappropriate and contributes to antimicrobial resistance. This study highlights the pressing need for an inte-grated approach to febrile illness management in rural Bangladesh, one that accounts for the changing epidemiology of infectious diseases, health seeking behaviour, and the limitations of the existing healthcare infrastructure. Expanding the ability of community health workers to manage patients with non-malarial febrile illnesses, stronger triage and referral systems, and addressing the financial and logistical barriers to care are critical steps toward improving health outcomes in these regions.

## 1 Introduction

Acute febrile illness remains a significant concern in tropical regions, where it is one of the most common reasons for seeking healthcare (Crump et al., 2017). In these regions, local populations often experience a high burden of infectious diseases, many of which present with fever as a primary symptom, including malaria and dengue, bacterial infections like typhoid fever, and acute respiratory infections (Prasad et al., 2015). Attempting to properly identify the cause of febrile illness is crucial, as delayed or incorrect diagnosis can lead to complications, prolonged illness, or death. However, case management is often difficult due to the non-specific presentation of symptoms, particularly early in the disease course (Crump et al., 2017; Grundy and Houpt, 2022). Beyond malaria diagnostics, rapid tests or laboratory assays for other diseases are not widely available in low-resource settings (Shrestha et al., 2018). As a result, patients may be misdiagnosed or managed with presumptive antimicrobial treatment, which is often ineffective and contributes to antimicrobial resistance (Karthikeyan et al., 2021; Ferdiana et al., 2021; Altaras et al., 2016; Rakotonandrasana et al., 2018). These issues tend to be more pronounced in remote and rural areas, where access to care is further constrained by geographical isolation, inadequate infrastructure, and shortages of skilled healthcare professionals (Scheil-Adlung, 2015). While clinical studies have documented these challenges in different countries (e.g., Edessa et al., 2024; Odaga et al., 2014), less attention has been paid to the broader context of care shaping practices and outcomes. Pathways to care, particularly in pluralistic health systems, are complex, diverse, and may involve consultations with different providers for the same episode of illness (Sundararajan et al., 2020). Each of these points of care can significantly influence the course of illness and patient management, which may be delayed or inappropriate. A holistic examination of the whole context of care and referral is therefore necessary to identify critical gaps, improve coordination, and develop strategies that can ensure timely, accurate, and effective services for all patients.

The current management and referrals system of acute febrile illness in Bangladesh offers a relevant setting to explore these issues. Over time, the national governments have expanded the public health infrastructure, developing an extensive network of health facilities and providers from the community level to tertiary hospitals, which co-exists with civil society organisations and a large and diverse private sector (MHFW, 2019; WHO, 2015). Despite significant progress in areas such as maternal mortality (El Arifeen et al., 2014), gaps in health service delivery remain against a backdrop of longstanding barriers to reaching poor ethnic communities in remote areas (Kabir et al., 2019). Malaria prevalence has declined, although it is still endemic in the Chittagong Hill Tracts (CHT) (Matin et al., 2020) along with continued or emerging public health issues associated with enteric fever, dengue, rickettsial disease and chikungunya (Das et al., 2022; Guenther et al., 2017; Hossain et al., 2023; Allen et al., 2024). One related concern is the large, and often inappropriate, use of antibiotics for the management of febrile illness, both in the public and private sectors (Samir et al., 2021; Rashid et al., 2022; Chowdhury et al., 2023; Sumon et al., 2024). In a recent hospital survey, for example, more than a quarter of patients who presented to outpatient departments for acute febrile illness had used antibiotics without prescription prior to admission, often reporting no improvement in their symptoms (Das et al., 2020).

In response to these challenges, the qualitative study presented here investigated how acute febrile illness is managed in Chattogram Division, Bangladesh, with a focus on practices and processes across different levels of the health system. By capturing the perspectives and lived experiences of the stakeholders involved, we identify good practices and gaps in service delivery, highlight potential areas for policy intervention, and contribute to strategies for improving healthcare access and quality in underserved areas.

## 2 Methods

### 2.1 Study design

The South and Southeast Asian Community-based Trials Network (SEACTN) is a large multidisciplinary project funded by the Wellcome Trust, which aims to define the burden of febrile illness in rural areas of Bangladesh, Cambodia, Laos, Myanmar, and Thailand, and assess interventions to mitigate health and economic impact (Chandna et al., 2022). In this qualitative study we contributed to the wider goals of SEACTN by exploring processes and practices for the management of acute febrile illnesses in Bangladesh, focusing on remote and rural areas in Chattogram Division, around Cox’s Bazar and the CHT. The research design was informed by concepts and methods in the social sciences, particularly studies of health seeking behaviour and pathways to care, which consider health care as a *process* involving multiple interactions with different healthcare providers (Kroeger, 1983; Broom et al., 2009; Sundararajan et al., 2020). We also considered the literature on health systems and policy analysis, which highlights the complexity of health service delivery within the key domains of access to human resources, medicines and technologies, information systems as well as the wider financing and governance frameworks (WHO, 2007, 2018). Data collection and analysis primarily involved qualitative interviews with stakeholders, allowing for a nuanced understanding of emerging issues, perspectives, and contextual influences. This approach is particularly valuable for exploring healthcare practices and processes, often shaped by social, cultural, and institutional dynamics that may not be evident through quantitative methods alone.

### 2.2 Study context

**Fig. 1:**
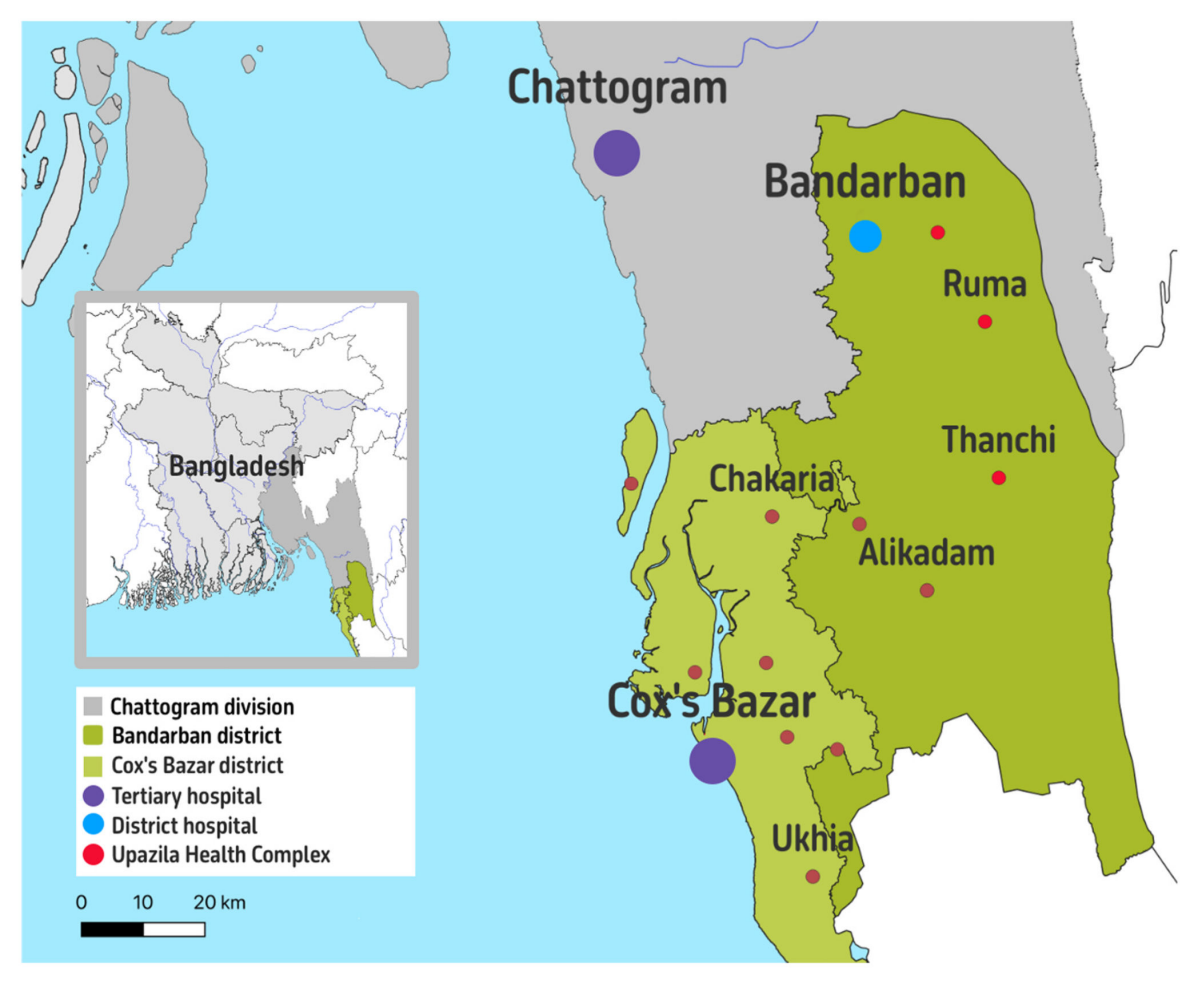
Study locations. Map generated using QGIS 3.4. National and provincial boundaries: Global Administrative Areas 4.1 (https://gadm.org). Health facilities: DGHS registry (https://hrm.dghs.gov.bd).

Chattogram is the largest administrative division in Bangladesh, notable for its diverse geography, which includes the coastal plains of Cox’s Bazar district, the rugged terrain of the Chittagong Hill Tracts, and the urban center of Chattogram city (Fig. 1). The region is home to various ethnic minorities, including the Chakma, Marma, Rakhine, Mro, and others. Each of these communities has its own distinct culture, languages, traditions, and health practices. In some remote areas, such as Bandarban district, they live in isolated conditions characterised by geographic inaccessibility and security issues stemming from the spill-over of the civil conflict from bordering Myanmar and separatist movements (Adnan 2022; Bal and Siraj, 2017). This has significantly impacted access to social services, including health care (Akter et al., 2019). The presence in Cox’s Bazar district of Rohingya refugees from Myanmar has further added to the region’s demographic and ethnic complexity (Bhatia et al., 2018).

The burden of infectious diseases in Chattogram is evolving and continues to present particular challenges in its hard-to-reach areas. The CHT has historically been one of the hotspots of endemic malaria in South Asia, shouldering 80 % of malaria cases in Bangladesh (WHO, 2023). Over time, malaria has declined considerably throughout the country although it remains endemic in the CHT, with additional concerns about the development of antimalarial resistance (Johora et al., 2021). Infectious diseases such as dengue (Mohapatra et al., 2022), tuberculosis (DGHS, 2020), rickettsial diseases (Kingston et al., 2018) and enteric diseases (Maude et al., 2016) are prevalent both in Cox’s Bazar and the CHT.

Responding to these challenges, the Ministry of Health and Family Welfare in Dhaka and the Health Services Division in Chattogram oversee an extensive network of public health facilities and services from the local communities to specialised urban hospitals, organised according to the tiered structure of the national health system and public administration, which consists of divisions at the top, followed by districts, upazilas (sub-districts), unions, and villages at the grassroots level:

◯ **Community clinics (CCs)**: Staffed by paramedics (Community Health Care Providers), these are the lowest-level public health facilities in rural areas, providing, among others, maternal and neonatal health care, integrated management for childhood illness, reproductive health and family planning services, the expanded programme on immunization, nutrition education and supplementation, health education and counselling, and acute care; each CC serves about 6000–8000 people
◯ **Union health and family welfare centres:** Staffed by nurses and, in some cases, doctors, they provide outpatient services only, focusing on maternal and child health and family planning
◯ **Upazila health complexes (UHCs)**: Located at the sub-district level, UHCs offer more comprehensive outpatient and inpatient services, including laboratory diagnosis for some infectious diseases (e.g., malaria, dengue, hepatitis, and enteric diseases), emergency care, and minor surgeries
◯ **District hospitals**: These serve as referral centres, offering more specialized care for non-communicable diseases, surgery, internal medicine, paediatrics, obstetrics, ophthalmology, and otorhinolaryngology
◯ **Tertiary hospitals**: Major cities have university hospitals, such as Chattogram Medical College, providing the most advanced and comprehensive services for infectious and non-infectious diseases, including intensive care units.

In addition to the public sector, the health system in Chattogram Division is diverse, involving actors in the private and NGO sectors, who fill gaps in health service delivery or respond to preferences in user demand. In urban and peri-urban areas, private hospitals and clinics play a significant role for both preventive and curative services. These facilities might offer better equipment and user-centred experiences, but they are typically more expensive compared to subsidized care in the public sector (Pavel et al., 2016). In rural areas, where traditional healers and informal providers are often the first point of contact, NGOs have established several programmes to improve access to care through outreach activities or community health workers (CHWs). The largest community-based programme is managed by BRAC, involving female health volunteers (*shasthya shebikas*) and their supervisors (*shasthya kormi*), reaching more than 100 million people across Bangladesh (Ahmed, 2008). In malaria-endemic areas, BRAC CHWs are supported by the Global Fund to Fight AIDS, Tuberculosis and Malaria to diagnose and treat malaria cases, refer severe cases to the nearest health facility, and other activities such as the distribution of bed nets and active case detection (Haque et al., 2010). For many years, BRAC CHWs have also been involved in the TB programme through awareness campaigns, screening, and referral of suspected cases (Paul et al., 2015).

### 2.3 Study participants

The study was conducted across multiple levels of the health system, from the central level of the Ministry of Health and Family Welfare to the local communities. Stakeholders were selected to represent a broad range of perspectives and experiences, including: (1) current and former senior managers responsible for planning and overseeing national health programmes in central departments of the Ministry of Health and Family Welfare in Dhaka and Chattogram city; (2) managers of community-based and vertical programmes in Dhaka, Cox’s Bazar district and Bandarban district in the CHT; (3) doctors, nurses, and paramedical staff at community clinics, upazila health centres and hospitals in Cox’s Bazar district and Bandarban district; (4) community health workers. Participants were recruited following a preliminary stakeholder mapping and using snowball sampling techniques to ensure diverse representation of both managerial staff and frontline health workers.

### 2.4 Data collection

Data were collected through semi-structured interviews conducted between June 2022 and August 2023 Each interview lasted between 30 and 60 minutes and was conducted either in person or via teleconference, depending on participant availability. The interview guide included open-ended questions designed to elicit detailed responses on the following topics: (1) perceptions about the burden and epidemiology of febrile illness in remote and rural communities; (2) the management of acute febrile illness, including diagnostic and referral practices; (3) perceptions about the effectiveness of the current health system in addressing these health issues; (4) prospects and recommendations for improving community care and the management of febrile illness. These broad interview topics were tailored to the distinct roles of participants, ensuring that questions were relevant to their expertise and experience within the health system. Interviews were audio-recorded with the participants’ consent and translated into English for analysis.

### 2.5 Data analysis

Data analysis followed a thematic approach (Braun and Clarke, 2006). The analysis of the pathways to care considered the key stages in clinical practice, including consultation, assessment, management plan, and referral. Transcripts were also coded inductively, with themes emerging directly from the data. The coding process was iterative, involving multiple readings of the transcripts to ensure that the themes accurately reflected the participants’ perspectives. NVivo 14 software was used to facilitate coding and data management. Two researchers independently coded the transcripts to enhance the reliability of the findings. Discrepancies in coding were resolved through discussion, and consensus was reached on the final themes.

### 2.6 Ethical considerations

The study received ethical approval from the Oxford Tropical Research Ethics Committee (Ref: 534–20) and the Institutional Review Board at the James P. Grant School of Public Health in Dhaka (Ref: IRB-31/12/21–049). All participants provided informed consent before participating in the study, with assurances of confidentiality and the right to withdraw at any time without any consequences. Data were anonymized during transcription, and identifying information was removed to protect participants’ privacy.

## 3 Results

In total we interviewed 35 stakeholders, as described in Table 1. The results are organized into several overarching themes, each reflecting the experiences, perceptions, and challenges faced by the study participants in relation to febrile illnesses and healthcare access. Emerging themes and anonymised citations are referenced by the unique identifiers in Table 1.

### 3.1 Health concerns in the communities

The study participants discussed their views and experiences about the most pressing health needs in rural communities, including infectious diseases, malnutrition, maternal and child health as well as the challenges related to service delivery and community engagement. When discussing the burden of malaria, health providers in Cox’s Bazar had tested only a few positive cases in recent months. However, malaria remained a concern in the CHT, with local health workers having observed a surge in malaria cases reflecting reports in the local news (Dhaka Tribune, 2022). A recurrent theme was the high malaria burden among ethnic minorities due to remoteness, health seeking behaviour, and cross-border transmission with neighbouring Myanmar. A manager of the malaria control programme in Cox’s Bazar, explained that the ongoing construction activities and the influx of migrant workers from the CHT had contributed to the continued presence of malaria in the region.

“Malaria has decreased here, but there are unions such as Tonkaboti where the incidence is still high. There are construction sites in these areas and the workers come from places where they do not use mosquito nets and personal protection” (Programme manager, Cox’s Bazar, M07).

“There is a tribe called Mro who live in the deep, mountainous part of Bandarban. They don’t like wearing long sleeves and use mosquito nets because they feel suffocated. The distribution of medicated net in those areas has failed” (Programme manager, CHT, M01)

“We might take steps to eliminate mosquitoes, but we share the border with Myanmar and we cannot stop malaria from there” (Medical officer, CHT, M04).

In many interviews across the health sector, dengue was mentioned as a growing concern, associated with increased population mobility between urban and rural areas. During one of our field visits, the country experienced one of the deadliest dengue outbreaks in recent history, thought to have originated in the densely populated Dhaka city, resulting in more than 200,000 dengue cases and 1000 deaths between January and September 2023 (Mahmud et al., 2024). A medical officer in Bandarban explained that an increase in tourists from Dhaka, attracted to the scenic hills and natural beauty of the region, had spread the disease to rural areas that were previously unaffected.

“In the past malaria was the most common diagnosis in the hilly areas, but recently the incidence of dengue has increased even in remote area like Royanchori, which is unusual. Tourists from Dhaka to Bandarban carry mosquitoes in airconditioned buses - this is how dengue is spreading. We need to keep in mind these causes of fever” (Medical officer, CHT, M04)

Several stakeholders also discussed the burden of water-borne diseases, particularly typhoid fever, mentioned in many interviews across the health sector as one of the most urgent priorities in rural areas. A university professor in Chittagong explained that the resurgence of water-borne diseases was associated with climate change and increased frequency of heavy rainfall and flooding, leading to the contamination of water systems with pathogens (C04). Other non-malarial acute febrile illnesses of particular concern are listed in Table 2.

### 3.2 Fever management

#### 3.2.1 In the communities

Pathways to care for febrile patients in the study locations varied, reflecting the diversity of the local health system, accessibility to health providers, cultural background, and costs. Health providers in both Cox’s Bazar and the CHT explained that the initial response to fever and related symptoms, such as stomach pain, typically involves the use of home remedies, traditional healers, or self-medication with antibiotics purchased in the communities at informal vendors or traditional healers themselves:

“Patients often rely on pharmacies for treatment, where the qualifications of pharmacists are sometimes questionable. These pharmacists frequently prescribe random antibiotics, and it is only when these treatments fail that patients seek hospital care. This practice not only leads to improper diagnoses but also increases the risk of developing antimicrobial resistance, making it more challenging to provide effective treatment when they eventually visit healthcare facilities” (Researcher, Dhaka, C09)

“[Medical assistants] in community clinics are not allowed to diagnose and prescribe antibiotic. Nonetheless, they are sometimes greedy (…) and sell antibiotics directly to patients to make a profit. Moreover, some pretend to be physicians, which is strictly prohibited by law” (Programme manager, Cox’s Bazar, M06)

“In many villages patients do not visit the health facility because they live far away … they would rather go to a village healer, who provides treatment without a proper diagnosis. They sell something like a cocktail therapy with a lot of medicines. This can cause problems such as antibiotic resistance” (Programme manager, Cox’s Bazar, M07)

If these initial approaches prove ineffective, patients and their caregivers may seek support from community-based health services. The public health value of both community clinics and CHWs was widely recognized by stakeholders across the health sector, who highlighted their contribution to malaria control, maternal health, and linking the communities with the formal health sector. It was also noted that CHWs are especially valuable among ethnic minorities and offer some advantages over Community Clinics, where high staff turnover and language barriers pose communication challenges (INT 03).

“When villagers have fever, they will seek treatment from traditional healers and are reluctant to seek treatment from doctors due to their customs and beliefs. This is a big problem in Bandarban. However, community health workers are more accepted by local people. They can encourage febrile patients to reach community clinics for further treatment and referral” (Medical officer, CHT, M03)

Some limitations of the current programmes also emerged. As malaria incidence has declined, the demand for healthcare has shifted to-wards other health concerns. In both the CHT and Cox’s Bazar, CHWs reported being approached frequently by patients seeking testing for non-malarial illnesses, such as dengue, typhoid fever, and chikungunya, as well as for diabetes and hypertension screening (P06, P08, P09, P11, P10). Due to their limited capacity and no guidance to manage these diseases, CHWs could only assist these patients by referring them to formal health centres for further testing and treatment.

“When the malaria test is negative, they ask for a typhoid test and paracetamol. But I have no other tests or medicines to provide” (Shasthya shebika, CHT, P06)

“They ask me if we do tests for typhoid, chikungunya, black fever, diabetes, blood pressure, and others. We tell them we can only test for malaria and they should see a doctor for the other diseases” (Shasthya kormi, CHT, P09)

“Those who travel to the hills are susceptible to malaria but malaria incidence is very low in the villages. Typhoid is the most prevalent disease in the villages now. The patients ask for typhoid tests and we tell we don’t have them. They ask for it every day” (Shasthya shebika, Cox’s Bazar, P11)

#### 3.2.2 Referrals

Participants recognized that the referral of patients who cannot be managed within the community remains an important public health function of CHWs and community clinics. However, access to public health centers, such as the UHC, and lower-level facilities such as the Union and Family Welfare Centres, can be challenging for the same reasons community services were initially established – long, uncomfortable travel and associated costs. In Bandarban, programme managers explained that villages in remote upazilas like Ruma and Thanchi can only be reached by trekking long distances, often requiring crossing of rivers. Road infrastructure is limited, and in extreme cases, “patients must be carried on other people’s backs” over tracts that are impassable by available vehicles (P07). A medical officer in Bandarban further explained that it could take five hours or even more than one day to reach the nearest health complex from remote villages because “the road is bumpy and transport is not available” (M10). In some places, the journey is further complicated by security issues, especially when traversing areas affected by conflict (C03). Consequently, “in the eyes of many villagers, health care begins and ends in the communities”, as a programme manager in Bandarban summarised (M02). Residents around Cox’s Bazar may have easier access to health facilities, although referral challenges were noted also in these areas due to inadequate ambulance services and lack of feedback from health facilities to confirm attendance of referred patients (P07, C01, M02, M03, M07). In some villages, the authorities managed a small social fund to pay for travel costs of referred patients (P01, P02) but we found no evidence such services were widely available in the study locations.

“If the malaria test is positive, the patient gets treatment, follow-up and usually gets better. If the malaria test is negative, the patient must go to the health facility. But they don’t like the burden of traveling, so they don’t go… Patients with fever expect their problems to be solved in the communities” (Programme manager, Cox’s Bazar, M07)

“Referring patients is hard due to poor networking between the CHWs and health facilities. There is no monitoring if the patient has reached the higher centres after referral, and this increases morbidity and mortality” (Senior health officer, Dhaka, C01)

“We should build more community clinics to reach the deeper parts of the hill tracks where road access continues to be an issue for villagers” (Programme manager, Bandarban, M03)

#### 3.2.3 Health centres and hospitals

If patients can travel outside their communities, they would often visit a private clinic. For those who cannot afford the higher costs in the private sector, subsidised health services are provided at the UHC, where doctors (“medical officers”), nurses, and a wider range of diagnostic and curative services are available for the management of febrile illness. Health workers at UHCs and central managers in Dhaka discussed progress made in health service delivery but were also conscious of persisting challenges such as insufficient availability of human resources, inpatient beds, and diagnostic/therapeutic capacities to meet demand (M07, M11, C08, C07). During one of our field visits, a UHC around Cox’s Bazar was crowded with patients, waiting in long queues in front of the wards. The doctors explained the health workforce was not enough to attend all visiting patients on the same day. Furthermore, limited laboratory capacity means that “many episodes of febrile illness remain undifferentiated and often treated with paracetamol and empirical antibiotics” (M03).

Another constraint is that intensive care units (ICUs) for patients in critical condition are typically not available at Upazila Health Complexes. District hospitals may have basic facilities to provide oxygen support, but are not yet equipped with advanced life-support technologies, such as ventilators or continuous monitoring systems necessary for managing more severe cases. In 2023, the local news reported that a project undertaken to set up ICUs in district hospitals had seen no progress after the launch in 2020 (The Daily Star, 2023). For those requiring more advanced care and diagnostics, therefore, referral to specialised tertiary hospitals, such as Chittagong Medical College is necessary, stretching travel times and costs even further. For example, traveling from Bandarban district hospital to Chattogram Medical College may take more than four hours by car, whereupon limited availability of ICU beds may further delay admission. One hospital manager commented that “this could be avoided if intensive care was provided at the upazila level” (C04).

“The Upazila Health Complex is not that far, only 4 km from Boat Bazar, but it is very far from Moricha or Inani. Patients feel discouraged to go there to seek treatment and ignore diseases like fever, jaundice, typhoid, and black fever. Diarrhoea is more prevalent now and one has to travel to the Sadar Hospital for diagnosis” (Programme manager, Cox’s Bazar, M09)

“The lack of sufficient diagnostic facilities at different levels in the healthcare system is a big issue for the management of febrile illnesses. Community clinics, union health centres, upazila health complexes do not have diagnostic facilities according to their jurisdiction and the number of patients they serve” (Senior health officer, Dhaka, C03)

“It is hard for the upazila health complex to treat patients in critical conditions because it is just a primary health care facility and there is no ICU support. If patients require ICU support, they must be transferred to a district or a tertiary hospital based on severity. And this takes time” (Assistant health director, Dhaka, C07).

### 3.3 Prospects

#### 3.3.1 Expansion of services

When discussing the future of community-based care, there was wide agreement that the provision of services could be expanded to address the evolving needs in the communities. Reflecting their views and experiences about health priorities, health workers and managers suggested that CHWs could be trained to perform rapid tests for non-malarial infectious diseases, and a broader range of WASH interventions to improve access to clean water and sanitation (M01). Many respondents highlighted the success of malaria RDTs, noting that similar tools could be used to test for common diseases such as dengue, typhoid, and chikungunya, with accompanying guidance to inform referral practices (P05, M03, M09). A technical officer in the WHO explained that increased diagnostic capacities would improve case management and reduce the high number of undifferentiated fevers:

“If a patient has fever and is negative for malaria, then the health workers don’t know what to do… this has been a problem for a long time. Such patients are classified as ‘pyrexia of unknown origin’. The focus at present is on malaria but other diseases like dengue and chikungunya need to be diagnosed and, for that, diagnostic tools are required. The WHO country office has recommended that community health workers should manage these diseases and raise awareness in their locations” (Technical officer at WHO, Dhaka, C02).

While there was general support for the introduction of new testing services, some expressed caution about using complex technologies at the community level such as multiplex RDTs that can detect different pathogens or disease markers. A researcher in Dhaka pointed out that “a misdiagnosis may cause problems or even antimicrobial resistance” (C05), suggesting that multiplex assays should be used at Upazila Health Complexes or NGO clinics, rather than being given to CHWs. A hospital manager noted that diagnostic tests are necessary only for a minority of patients, who cannot be diagnosed through symptoms and physical examination:

“Fever is not a disease, but rather a symptom and the disease needs to be diagnosed be it viral or protozoal disease. However, I can say that 70–90 % of patients in this hospital can be diagnosed only by symptom identification and physical examination and only 10–20 % of patients require laboratory investigation to confirm or rule out diagnoses” (Hospital manager, Chattogram, C04)

#### 3.3.2 Innovation

The potential of digital technologies was acknowledged, especially for improving disease surveillance and streamlining data collection. Many healthcare providers were already familiar with digital health applications, used in the communities to report cases during the COVID-19 pandemic (P02). Others suggested that digital systems could be used to strengthen the referral system by checking if patients have reached the referral destination:

“Whenever a patient is referred to a higher-level health center, the community health worker can report to the medical officer so they can check if the patient has reached the facility and treatment has started” (Senior health officer, Dhaka, C01)

Challenges related to poor mobile network coverage in remote areas were also mentioned (C01), while some were more sceptical about the ability of community health workers to use digital tools, citing the need for regular monitoring and adequate training (M08).

#### 3.3.3 Financing

Despite wide recognition of opportunities for innovation and further development of community-based care, there was acknowledgement that any expansion of responsibilities would need to be sustained by an increase in resources and support. Low remuneration for CHWs was a recurrent concern, with many respondents arguing that they could not sustain a livelihood, resulting in high attrition rates. Most CHWs agreed that an increase in salary would significantly improve their motivation and capacity to handle additional responsibilities.

“It will be fine if you give us an extra salary. We now earn 4000–5000 taka [US$ 30–40] which is not enough. If you increase our salary, then we won’t have any problems doing this. If we are taught practically, then we will be able to do it. It will be better if you provide us with some transportation allowance” (Shasthya shebika, Cox’s Bazar, P11)

Furthermore, CHWs were already overwhelmed with various responsibilities, including regular household visits and participation in vaccination campaigns (C07, M04). The burden of home visits was even greater in remote areas, where “travel between households can take an hour” (INT 02). Thus, many respondents emphasized that expanding health programs—such as adding new tasks or services for CHWs—would not be feasible without a substantial increase in resources and workers. A health manager in Dhaka underscored this point by drawing a parallel with academic work:

“Increased workload would result in a lower quality of care and we do not want that. Suppose we ask you to write ten papers in a month and we will provide you with 20,000 Taka instead of 10,000 Taka or even more money. You may do the work but the quality will be lower. We want to provide high-quality services so we need more health workers in the communities” (Assistant health director, Dhaka, C07)

The allocation of additional resources for programme development, however, was described as a significant challenge in most interviews with managers and planners, particularly as donor funding -which many programs rely on - has been declining. A program manager explained that the government lacks the financial resources to fully fund and operate the entire health system on its own. As a result, the involvement of donor agencies was seen as critical to support both NGOs and government agencies in delivering essential healthcare services (M07). Considering these issues, it was suggested that the introduction of small user fees for services in the communities, such as diagnostic tests, could be a potential solution to improve the system’s ability to fund itself. A BRAC executive further commented that a small fee might also increase the perceived value of CHWs in the communities due to the belief that services provided for free are often of lower quality (C08).

## 4 Discussion

This qualitative study explored challenges and opportunities for the management of acute febrile illness in rural and remote areas of Chattogram Division, Bangladesh. By engaging with stakeholders across the health sector, the study identified critical themes related to healthcare access and potential improvements. The findings reflect both the strengths of the current health infrastructure and persistent gaps that hinder the effective management of febrile illness and other health issues, particularly in underserved areas.

While malaria has declined, the disease persists in areas where the environment, cultural resistance to preventive measures, and cross-border transmission are seen as key drivers of disease transmission. Concurrently, dengue and other diseases such as typhoid fever have emerged as a growing concern (Chew et al., 2022). The control and management of these infections is further complicated by socio-cultural factors, particularly among isolated groups who live in precarious conditions and are disconnected from public health services (see also Matin et al., 2020).

As described, CHWs and community clinics are recognized as essential care providers in these settings for their multiple roles in malaria prevention and treatment, TB control, maternal and child health support, family planning, and, recently, COVID-19. However, the findings indicate that the evolving health needs of the communities have outpaced the current capacity of CHWs, who are increasingly consulted for non-malarial illness. This mismatch between community demand and the skills of CHWs has been documented in other countries (Glenton et al., 2013; Liverani et al., 2017, 2024), highlighting the need for differential diagnosis and management of febrile illnesses beyond malaria (Kalyango et al., 2012; Guenther et al. 2017; McLean et al., 2018; Nguyen et al., 2024; Orng et al., 2024). One option to meet this need is the expansion of community-based services through new testing technologies. In particular, lateral-flow assay tests for non-malarial diseases, including dengue RDTs and combination test kits capable of detecting multiple pathogens, are increasingly available in the market (Ince and Sezgintürk, 2022; Pollak et al., 2023). Another option is the introduction of disease severity markers, such as endothelial and immune activation markers, into existing malaria RDTs. These markers can predict the pathogenesis of severe and fatal infections and therefore could be used to risk-stratify patients and inform management, regardless of aetiology (McDonald et al., 2018). Despite concerns about the feasibility of more complex assays for CHWs in some interviews, these tests offer a practical and scalable solution for rural deployment due to their affordability, ease of use, and minimal infrastructure requirements. In Bangladesh, expanded testing services in community settings could enhance patient triage reducing unnecessary travel to health facilities and unnecessary antibiotic dispensing (Chandna et al., 2021). They would also be useful in health centres and hospitals, where empiric diagnosis and treatment of acute febrile illness based on symptoms alone seem common, despite the wide range of pathogens. Additionally, these tests could be integrated with electronic decision support tools, further assisting health workers in making consistent treatment and referral decisions (Pellé et al., 2020). Recent studies in Bangladesh and other countries have documented the value of digital technologies for community-based care and the response to COVID-19 (e.g., Martin et al., 2020; Patwary and Islam, 2025). However, evidence about the effectiveness of such technologies for undifferentiated febrile illness management remains inconclusive and will require more findings from ongoing and future investigations (e.g., Chew et al., 2024). Implementation of any expanded services should also address carefully financing issues, ensuring adequate compensation for CHWs. As seen in our study, many participants expressed frustration over low remuneration and high workload, reflecting global concerns about widespread unpaid labour among CHWs (Wennerstrom and Smith, 2023).

Lastly, our study documents significant referral challenges across the health sector, exacerbated in critical patients who undergo multiple transfers before reaching the level of care they need. As a result, they may arrive too late or never reach the destination of care. In the most remote regions, emergency teams stationed at community clinics could be established, with the necessary equipment and ambulance-converted vehicles for reaching and helping these patients access roads and health facilities (Hofman et al., 2008). In areas with network coverage, digital health solutions offer some promise (Marino et al., 2024). For example, there is increasing interest in the deployment of smart ambulance services to optimise both the location and routing of emergency response vehicles in low-resource settings (Boutilier, et al., 2020). In Bangladesh, there is a favourable policy environment to support these innovations, particularly the national Digital Health Strategy, which emphasises the need for “improvements in information sharing in urgent and emergency care widening access to telehealth services, especially in rural and remote Bangladesh” (MoHFW 2023: 33). However, for these to be feasible, better tools for risk stratification and triage are necessary, as discussed, in order that unnecessary referrals do not place additional strain on already overburdened health systems.

To inform these and other possible interventions, further research is needed. The narrative presented here provides an overview of challenges and opportunities through the *viva voce* of the stakeholders involved but cannot provide a quantitative assessment of these processes or the disease epidemiology. Future studies, for example, could estimate the proportion of missed referrals at various stages in the care pathways and key indicators such as the number of misdiagnoses or inappropriate treatment of febrile patients, and their contribution to mortality. Furthermore, in addition to health providers and managers, understanding the views and experiences of private providers, traditional healers, and community members themselves would provide a more comprehensive mapping of pathways to care and the broader socio-cultural context influencing febrile illness management. It should also be acknowledged that data collection was partly conducted when COVID-19 restrictions were still in place, with constraints to field work and the availability of informants for interview.

## 5 Conclusion

This study highlights the urgent need for a comprehensive strategy to manage febrile illnesses in remote and rural Bangladesh - one that accounts for evolving patterns of infectious diseases, healthcare-seeking behaviours, and the constraints of the current health system. Key priorities include enhancing the capacity of community health workers to treat non-malarial fevers, strengthening triage and referral mechanisms, and overcoming financial and logistical obstacles to accessing care. Given the increasing availability of low-cost technologies that can be used to diagnose and manage acute and febrile patients at point of care, there are new opportunities to strengthen frontline health services and improve timely access to appropriate treatment. For these innovations to be effective, they must be carefully adapted to their contexts of implementation, considering the infrastructure and the constraints faced by primary care providers. It is equally important to ensure the sustainability of these innovations by integrating them into the wider health system, with careful attention to capacities, long-term financing, and alignment with the needs and preferences in the communities.

## Figures and Tables

**Table 1:** Interview participants.

Directors/deputy directors of health departments or institutes of the Ministry of Health and Family Welfare, Dhaka and Chattogram city	C01, C03, C04, C05, C07, C09
Managers of NGO community programmes, Dhaka	C06, C08
Programme managers and medical officers in Cox’s Bazar district	M02, M05, M06, M07, M09, M11
Programme managers and medical officers in Bandarban district, CHT	M01, M03, M04, M08, M10
Community health workers at community clinics or BRAC in Cox’s Bazar district	P05, P08, P10, P11, P12
Community health workers at community clinics or BRAC in Bandarban district, CHT	P01, P02, P03, P04, P06, P07, P09
Stakeholders in international organisations	C02, INT01, INT02, INT03, INT04

**Table 2:** List of non-malarial diseases mentioned in the interviews.

Viral diseases	
Dengue fever	C02, C04, C05, C06, C07, INT03, M02, M03, M04, M06, M07, M08, M09, M10, M11, P01, P06
Chikungunya fever	C02, C04, C05, C06, M06, M09, M10, M11, P09, P10, P11
Zika	C06
**Bacterial diseases**	
Leptospirosis	C04, C06, M06
Meningitis	C04
Pneumonia	P06, P07
Rickettsial disease	C04, C06, M06
Typhoid/enteric fever	C02, C05, C04, C06, C07, C08, M02, M06, MO7, M08, M09, M10, P01, P02, P04, P05, P06, PO7, P08, P09, P10, P11, P12
